# Decoding the Role of Caveolin‐1 in Morphological Diversity and Self‐Renewal of Breast Cancer Cells

**DOI:** 10.1111/cpr.70137

**Published:** 2025-10-30

**Authors:** Shun Li, Hongyun Duan, Lu Yang, Lingyi Jiang, Haocheng Bian, Yuqin Jiang, Yixi Zhang, Wei Yan, Qin Yang, Tingting Li, Xiang Qin, Zong‐Yuan Liu, Ningwei Sun, Kai‐fu Yang, Yiyao Liu

**Affiliations:** ^1^ Department of Oncology & Cancer Institute, Sichuan Academy of Medical Sciences, Sichuan Provincial People's Hospital, and School of Life Science and Technology University of Electronic Science and Technology of China Chengdu Sichuan China; ^2^ MOE Key Laboratory for Neuroinformation, School of Life Science and Technology University of Electronic Science and Technology of China Chengdu Sichuan China; ^3^ Department of Engineering Mechanics, Institute of Biomechanics and Medical Engineering Tsinghua University Beijing China; ^4^ Traditional Chinese Medicine (TCM) Prevention and Treatment of Metabolic and Chronic Diseases Key Laboratory of Sichuan Province Hospital of Chengdu University of Traditional Chinese Medicine Chengdu Sichuan China; ^5^ Department of Urology Deyang People's Hospital Deyang Sichuan China

**Keywords:** cancer cell morphology, caveolin‐1, cytoskeleton rebuilding, focal adhesions, self‐renewal ability

## Abstract

Cellular geometry is tightly associated with the function of a cell. During tumour progression, cancer cells undergo changes in phenotypes and biological behaviour with deformations in cellular morphology. However, whether the morphological diversity of cancer cells correlates with the cellular phenotype, and the underlying mechanism of morphology‐related function in cancer cells is still unclear. Here, we simplified the cellular morphology by clustering cancer cells into three categories based on two‐dimensional cellular morphological features. The silence of caveolin‐1 (Cav‐1), the primary constituent of membrane caveolae, reproduced the morphological evolutionary behaviour of cancer cells, which is similar to the epithelial‐mesenchymal transition process. The attenuation of dorsal stress fibres, the assembly of focal adhesions and the disorder of transverse arc fibres and their regulatory signals are demonstrated as the main morphological evolutionary tools of cancer cells. Moreover, a modified vertex model theoretically reconfirmed the evolutionary process of cellular morphology. Small GTPases and focal adhesion kinase signalling were implicated in Cav‐1 knockdown‐induced cytoskeletal remodelling and focal adhesion assembly. Both in vitro and in vivo studies have demonstrated that Cav‐1‐dependent morphological changes are closely associated with the self‐renewal capacity of breast cancer cells. Overall, our work highlights new insight into the morphological diversity and the correlation between cellular shape and phenotype of cancer cells, and provides evidence that Cav‐1 could affect cancer cell properties such as self‐renewal capacity through maintaining the morphological stability.

## Graphical abstract

The silence of caveolin‐1 (Cav‐1) reproduced the morphological evolutionary behaviour of cancer cells, which is similar to the epithelial‐mesenchymal transition process. And Cav‐1 dependent morphological changes could affect cancer cell self‐renewal capacity through maintaining the morphological stability.

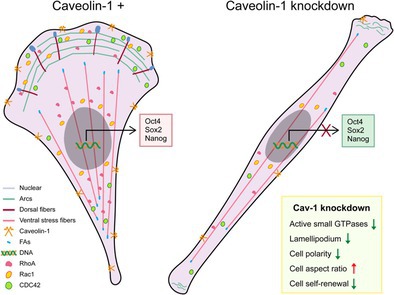

## 1 Introduction

The geometric morphology of cells in an organism is tightly regulated, while remodelling of cellular morphological features usually implies changes in cell phenotype and function [1, 2]. Both stabilisation and dynamics of cellular morphology are indispensable. Morphological dynamics fulfil the need for cellular movement or the acquisition of new cellular functions, whereas terminally differentiated cells could maintain tissue organisation and function through relatively static morphology. For example, during the process of embryogenesis, biochemically and mechanically regulated epithelial cells undergo morphological remodelling for the construction of new tissue structures [3]. After development, stabilised epithelial cell morphology then maintains the physical structure of tissues or organs. More importantly, cellular morphological plasticity is one of the most important regulatory factors in the pathogenesis of diseases such as cancer [4, 5]. For instance, during carcinogenesis, epithelial cells remould their morphology and tissue properties to accommodate directional migration, invasion or proliferation through epithelial‐mesenchymal transition (EMT) [6, 7]. Therefore, the morphological diversity of tumour cells may largely reflect the fate of the cancer [8], and elucidating the morphological evolution of tumour cells and related regulatory mechanisms may provide novel strategies for cancer diagnosis and treatment.

In the physiological niche, the geometry of cells is determined by the spatial constraints imposed by the extracellular matrix (ECM) and/or other surrounding cells [9, 10]. Thus, changes in physical connections and interactions between cell–cell and cell–ECM usually lead to alterations in cell morphology and phenotype [11]. During tumorigenesis, the loss of cell–cell or cell–matrix adhesion initiates the process of EMT, which is characterised by changes in cell morphology, increased cell motility, proliferation and heightened drug resistance, all of which contribute to the tumour progression [12, 13, 14]. In addition, changes in intrinsic genetic or biochemical signalling can also lead to the reconstruction of cell morphology through remodelling of the cytoskeletal network [8].

Caveolin‐1 (Cav‐1) is a structural protein necessary for cell membrane invagination to form a niche structure, with a molecular weight of about 22 kD, which has a transmembrane hairpin structure, played roles in a variety of physiological functions including endocytosis, organisation of the ECM, cholesterol distribution, cell migration and signalling [15]. Over the past two decades, there has been growing evidence that Cav‐1 plays a central regulatory role in many diseases such as cancer [16], diabetes [17] and fibrosis [18]. Cav‐1, as a mechanosensor, is involved in the regulation of the properties and behaviours of tumour cell skeleton remodelling [19], migration [20], invasion [21], apoptosis [22] and metabolism [23]. High levels of Cav‐1 in various tumour types are positively correlated with tumour growth, metastasis and drug resistance, leading to accelerated tumour progression and poor clinical outcome [24]. Therefore, considering that Cav‐1 has a significant targeted regulatory effect on the regulation of tumour cell morphology and tumorigenesis, we would like to use Cav‐1‐dependent morphological remoulding in breast carcinoma as a model to explore the evolutionary pattern of tumour cell morphology and its impact on the biological behaviour of tumour cells.

Here, we analysed the morphological diversity of tumour cells and clustered the morphology of MDA‐MB‐231 cells by using the *k*‐means clustering algorithm method and the cellular shapes were classified into three categories, and the morphological features show characteristics of continuous evolution. The expression level of Cav‐1 is closely related to the morphological features such as the aspect ratio and the width of the lamellipodium leading edge. Further, we confirmed that Cav‐1 is indispensable in the remodelling of cell morphology by adjusting the cytoskeletal network, especially the actin networks, which are strongly associated with the function and characteristics of the tumour cells. Moreover, vertex mechanistic modelling of single cells also confirms that cellular actin networks and focal adhesions (FAs) contribute to the evolution of cell morphology with a continuous transition phenotype. We further demonstrated that cell morphology can directly influence the self‐renewal phenotype of tumour cells by controlling tumour cell spreading area and shape through micropatterning. Together, we reported the Cav‐1‐dependent evolution of cell morphological features and its relationship with the self‐renewal ability of cancer cells, which could provide potential strategies for cancer diagnosis and treatment based on the graphics and biomechanics.

## 2 Materials and Methods

### 2.1 Cell Culture

MDA‐MB‐231 cells were purchased from the Cell Bank of Type Culture Collection of Chinese Academy of Sciences (Shanghai, China), and shCav‐1 MDA‐MB‐231 cells were obtained in our earlier study [25]. Cells were cultured in Leibovitz's 15 cell culture medium (Gibco, USA). The growth medium was supplemented with 10% foetal bovine serum (FBS, Gibco) and 1% penicillin (Gibco). For shCav‐1 MDA‐MB‐231 cells, the culture medium was additionally supplemented with 0.5 μg/mL puromycin (Gibco). Cells were maintained in a standard humidified incubator at 37°C.

### 2.2 In Vivo Animal Studies

To investigate the effect of Cav‐1 on tumour self‐renewal, 5‐week‐old female NCG severe immunodeficiency mice were purchased from GemPharmatech (China), and before tumour cell injection, mice were acclimated for 1 week under specific conditions. Ten mice were subcutaneously injected with 100 μL of Matrigel (Corning, USA) containing 1 × 10^6^ MDA‐MB‐231 cells or shCav‐1 MDA‐MB‐231 cells. Each group consisted of five mice. The experiment was terminated at the 7th week (three mice per group) and the 9th week (two mice per group). Tumour volume was measured using callipers, and tumour weight was recorded. Immunohistochemical experiments were performed on tumour sections. Tumour volume approximated as: tumour volume = (tumour width × tumour length × tumour height) × *π*/6. The animal protocols complied with animal welfare laws and were authorised.

### 2.3 Immunofluorescence Microscopy

For immunofluorescence procedures, the cells were fixed in 4% paraformaldehyde for 15 min, permeabilised with 0.4% Triton‐100 for 10 min and blocked with 1% BSA at 4°C for 12 h. The primary antibodies for immunofluorescence were applied at 4°C for 12 h. Alexa488, Alexa555 and Alexa647‐labelled secondary antibodies were obtained from Jackson ImmunoResearch. Alexa555 and Alexa647 conjugated phalloidins were obtained from Abcam (USA). Images were captured using an Eclipse Ti2 microscope (Nikon) and an LSM 810 confocal microscope (Zeiss, Germany).

### 2.4 Western Blotting

After washing the cells with pre‐chilled PBS, RIPA (Beyotime, China) containing protease and phosphatase inhibitors was added. The protein content was determined using a BCA protein concentration assay kit (Beyotime), and the samples were mixed with sample buffer, boiled and run on 10% or 12.5% SDS‐PAGE gels (Bio‐Rad, USA). After separation, the proteins were transferred to a PVDF membrane, blocked and incubated with primary antibodies overnight at 4°C. Subsequently, secondary antibodies were added and incubated at room temperature for 2 h. Protein detection on the membrane was performed using an ECL chemiluminescence assay kit (BeyoECL Star, Beyotime), and protein bands were quantitatively analysed using Fiji ImageJ.

### 2.5 Antibodies

Primary antibodies used: phospho‐cofilin (Ser3), rabbit monoclonal, CST 3313; Cav‐1, rabbit monoclonal, CST 3238; phospho‐myosin light chain 2 (Thr18/Ser19), rabbit monoclonal, CST 95777; Oct‐4A, rabbit monoclonal, CST 2890; Nanog, rabbit monoclonal, CST 4903; Sox2, mouse monoclonal, CST 4900; Profilin 1, rabbit monoclonal, Abcam ab124904; Vinculin, rabbit monoclonal, Abcam ab129002; Cofilin, rabbit monoclonal, Abcam ab124979; Arp3, rabbit monoclonal, Abcam ab181164; FAK (phospho Y576), rabbit monoclonal, Abcam ab76120; F‐Actin, rabbit polyclonal, Absin abs123596; RhoA, rabbit monoclonal, Beyotime AF2179; CDC42, rabbit monoclonal, Beyotime AF2794; RAC1, rabbit polyclonal, Beyotime AF7854; beta Actin, rabbit polyclonal, Servicebio GB11001. Secondary antibodies used: Alexa Fluor 488, Alexa Fluor 555 and Alexa Fluor 647 (Jackson ImmunoResearch). Others: Phalloidin‐iFluor 555 (Abcam, ab176756); Phalloidin‐iFluor 647 (Abcam, ab176759).

### 2.6 Cell Segmentation and Statistical Analysis of Single Cells in Microscopic Images

We first segment each cell with a well‐trained deep learning‐based segmentation method, that is, Cellpose, which can precisely segment cells without further parameter adjustments [26]. Subsequently, six shape‐related morphological features were utilised to describe the shape of the cells. Specifically, six features of shape used in this study include (1) roundness, to measure how closely the shape of a cell approaches that of a circle, (2) extent, defined as the ratio of pixels in the cell regions to pixels in the total bounding box, (3) inscribed circle ratio, defined as the ratio of pixels in the maximum inscribed circle to pixels in the cell region, (4) aspect ratio, defined as the ratio between the lengths of the major and minor axes of the ellipse that has the same normalised second central moments as the region, (5) symmetric ratio, defined as the ratio of the area of the non‐overlapping portion resulting from folding the cell region about its minor axis to the cell's area and (6) signature peaks, defined as the number of peaks in the signature that represents a one‐dimensional function, that is, the distance from the centroid to the boundary served as the function of angle.

The obtained morphological features were first normalised, and then clustered into three groups using the typical *k*‐means clustering algorithm [27]. Based on the results of clustering, we found significant shape differences existing among the cells in three groups. For example, cells in the first group have roughly circular shapes, while cells in another group are characterised by elongated, spindle‐like shapes. Cells in the third group exhibited a morphology of droplet shapes. Finally, we employ the t‐distributed Stochastic Neighbour Embedding (t‐SNE) method, a widely used dimensionality reduction technique frequently utilised for high‐dimensional data visualisation [28], to visualise the distribution of shape features of cells in each group.

### 2.7 Focal Adhesion Analysis

After the immunofluorescence staining of vinculin and microscopic imaging, each FA was identified and manually outlined based on its distinct morphology and fluorescence intensity relative to the background. The number of FAs per cell was counted after the manual identification and segmentation. The area of each manually outlined FA was calculated using the measurement tools in ImageJ.

### 2.8 Real‐Time Quantitative PCR

RNA was extracted from cell samples using the FastPure Cell/Tissue Total RNA Isolation Kit (Vazyme, China). cDNA was synthesised from the purified mRNA using HiScript III RT SuperMix for qPCR (Vazyme) according to the manufacturer's instructions. The cDNA was amplified using Taq Pro Universal SYBR *q*PCR Master Mix (Vazyme) with primers and β‐actin was used as a housekeeping gene. Results were calculated using the equation ^ΔΔ^ *C*_T_ and expressed as a multiple of change from control.

### 2.9 Fluorescence Resonance Energy Transfer (FRET) Analysis

Transfection of cells was performed using Lipofectamine 3000 transfection reagent (Thermo Fisher Scientific, USA) with FRET plasmids (Rac1, CDC42 and RhoA). After transfection for 8 h, the culture medium was replaced with fresh complete medium, and the cells were further incubated for 24 h. Fluorescence imaging was conducted using an Eclipse Ti2 microscope (Nikon, Japan). Fluorescence images in both the YFP and CFP channels were acquired, followed by analysis of the ratio map using ImageJ software to calculate the fluorescence ratio (YFP/CFP).

### 2.10 Modified Vertex Model for the Cell Morphology Evolution

In this study, we modified the biophysical model that we have developed previously [29] to understand the Cav‐1‐dependent morphological evolution of cancer cells. A modified vertex model is generated to reproduce three representative morphologies experienced in the morphological evolution of cancer cells. Here, the number of vertices was increased to characterise the shape changes of cancer cells with large deformations and high dynamics and the cell shape changes were determined by the dynamics of vertices distributed along the cell basal membrane, which follow the force equilibrium,

(1) $$\eta_{\mathrm{Vis}}\frac{dx}{\mathrm{dt}}=F_{\text{Tension}}+F_{\text{Area}}+F_{\mathrm{Rac}}+F_{\text{adhesion}}+F_{\text{Fibre}}+F_{\text{Noise}}$$

where t is time, x is the position of the vertex and $\eta_{\mathrm{Vis}}$ is the drag coefficient resulting from the tissue viscosity. Multiple forces on vertices have been considered here: $F_{\text{Tension}}$ is the cortical tension, $F_{\text{Area}}$ is the area stiffness of cell resulting from the internal osmatic pressure, $F_{\mathrm{Rac}}$ is the Rac1‐induced protrusion force, $F_{\text{adhesion}}$ is the cell‐matrix adhesion force, $F_{\text{Fibre}}$ is the stress of stress fibres and $F_{\text{Noise}}$ is the Gaussian white noise arising from thermal fluctuations. The governing equation is numerically integrated using the finite difference method with a time step Δt.

For a single vertex,

(2) $$F_{\text{Tension}}=K_{L}s^{\pm }$$

where $K_{L}$ is the line tension alone the cell cortex and $s^{\pm }$ is the vector pointing to the neighbouring vertices.

(3) $$F_{\text{Area}}=-K_{a}\hat{s}\left(A-A_{0}\right)r_{\mathrm{nor}}$$

where $K_{a}$ is the areal elastic modulus of the cell, $\hat{s}=\frac{\left(\left|s^{+}\right|+\left|s^{-}\right|\right)}{2}$ is the coverage cortical length of the current vertex, $A-A_{0}$ is the change of cell area during morphogenesis, $r_{\mathrm{nor}}$ is the normal direction of the local cell cortex.

(4) $$F_{\mathrm{Rac}}=f_{\mathrm{Rac}}\frac{1}{\sigma \sqrt{2\pi }}e^{-\frac{1}{2}\left(\frac{2t-T}{2\sigma }\right)}r_{\mathrm{nor}}$$

where $f_{\mathrm{Rac}}$ is the normal strength of Rac protrusion, σ is the standard deviation (SD) of Rac strength within one Rac cycle, T is the duration of the activated Rac cycle and t is the current morphogenesis time.

(5) $$F_{\text{adhesion}}=f_{\text{CAMs}}\frac{{L_{\mathrm{Rho}}}^{n}}{K_{L}+{L_{\mathrm{Rho}}}^{n}}\cdot \mathrm{\Delta x}$$

where $f_{\text{CAMs}}$ is the adhesion strength of CAMs and $L_{\mathrm{Rho}}$ is the normal concentration of RhoA GTPase, $K_{L}$and n are the apparent dissociation constant and the Hill coefficient, respectively.

(6) $$F_{\text{Fibre}}=K_{\text{Fibre}}\left(L-L_{0}\right)$$

where L is the current length of the stress fibres and $L_{0}$ is the critical length of the stress fibres.

(7) $$F_{\text{Noise}}=f_{R}r_{\text{Noise}}$$

where $r_{\text{Noise}}$ is the unit‐variance Gaussian noise vector at the current vertex and $f_{R}$ is the strength of the Gaussian white noise.

### 2.11 Statistical Analysis

All data are presented as mean ± SD. GraphPad Prism software (USA) was used to compare results between two groups by the unpaired Student's *t* test and one‐way ANOVA test with Tukey's post‐test analysis was used when comparing multiple groups (GraphPad Prism). Differences were considered statistically significant when *p* < 0.05 (*), *p* < 0.01 (**) or *p* < 0.001 (***), while differences were considered remarkably statistically significant when *p* < 0.0001 (****).

## 3 Results

### 3.1 Morphological Diversity of Breast Cancer Cells

**FIGURE 1:**
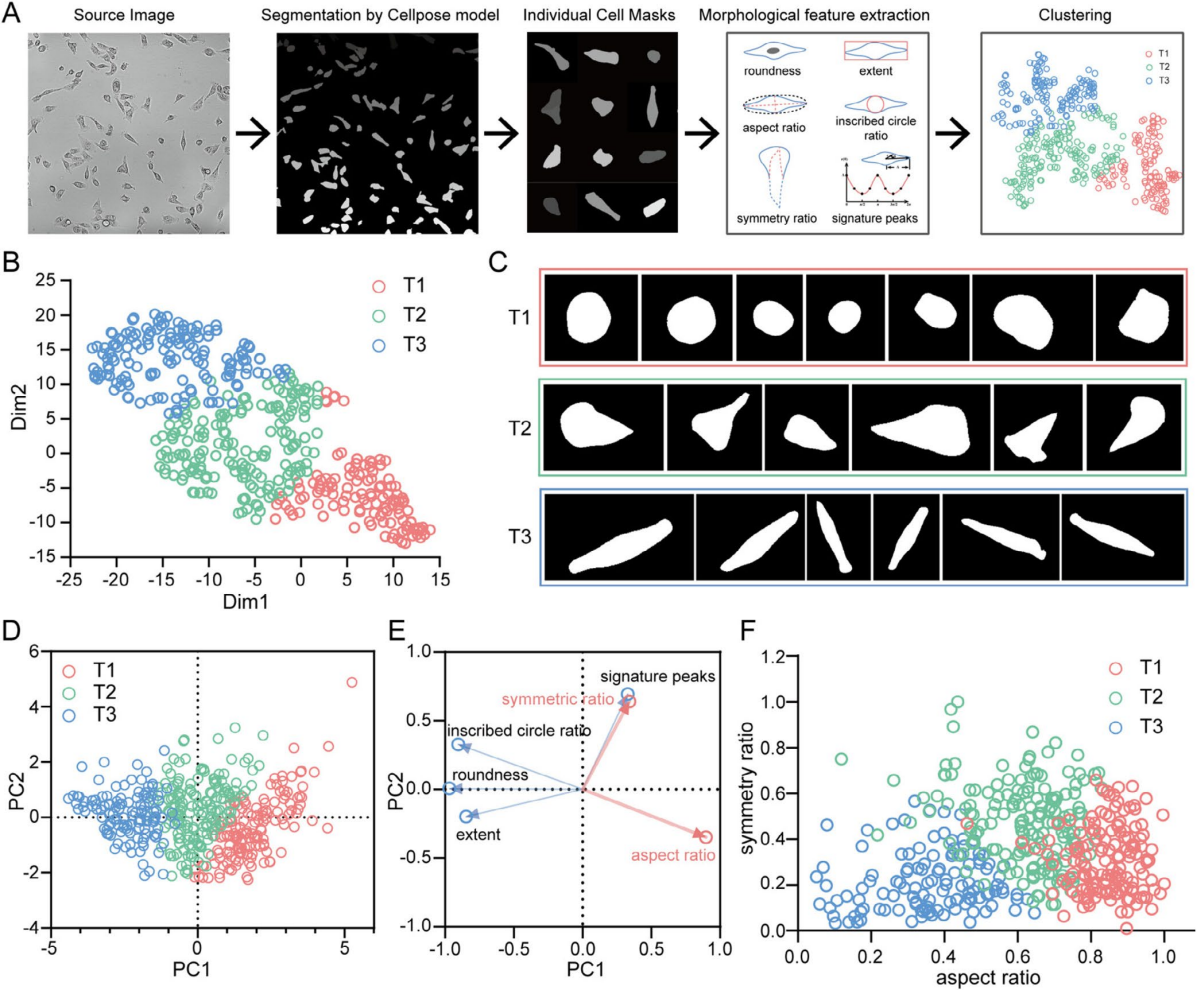
Morphological diversity of breast cancer cells. (A) Schematic diagram of cell clustering process. (B) Dimensionality reduction of morphological clustering results to visualise by t‐SNE. (C) Representative cell shapes from dataset of three types. (D) Principal component analysis of cell shapes using traditional shape descriptors of roundness, extent, inscribed circle ratio, aspect ratio, symmetric ratio and signature peaks. (E) The vectors describing the morphospace demonstrate how each shape descriptor contributes to the first two components of PCA. (F) Scatter plot illustrates the variations in cell shape across the dimensions of symmetry ratio and aspect ratio. (B, D and F) *n* = 432.

Given the significant impact of alterations in cellular morphology on the function and behaviour of tumour cells, we first evaluated the morphological heterogeneity of MDA‐MB‐231 cells, the representative triple‐negative breast cancer cell line, on a 2D surface. To extract morphological images of single cancer cells, we utilised the Cellpose algorithm to perform cell body segmentation on microscopic images of MDA‐MB‐231 cells (Figure 1A). To quantify the cell shape, after the segmentation, morphological descriptors including circularity, minimum enclosing rectangle area ratio, aspect ratio, peak number, maximum inscribed circle ratio and symmetry ratio (Figure 1A) were used to characterise the cell morphology. After normalising the data representing and analysing the morphological characteristics of cancer cells, a *k*‐means clustering algorithm was employed to categorise the cellular shapes into three distinct clusters. When the clustering results were examined, it was evident that there were significant morphological differences between the three cell type categories (T1, T2 and T3): Cluster T1 showed predominantly round‐shaped cells, T3 was characterised by elongated spindle‐like cells and T2 exhibited an intermediate morphology reminiscent of droplets (Figure 1B,C). Notably, sub‐cluster T2 exhibited transitional features between the round‐shaped T1 and spindle‐like T3 categories (Figure 1B). To reduce the number of variables needed to describe the cell morphology, the shape descriptors were subjected to principal component analysis (PCA) (Figure 1D). Among the factors, aspect ratio is a more classical cellular shape descriptor that significantly impacts PC1, while the almost geometrically orthogonal symmetry ratio has a large influence on PC2 (Figure 1E). Therefore, the aspect ratio and the length of leading edge (a biological description of symmetry ratio) were chosen for subsequent experiments to characterise the cellular morphology of cancer cells (Figure 1F).

### 3.2 Cav‐1‐Dependent Cancer Cell Morphology

**FIGURE 2:**
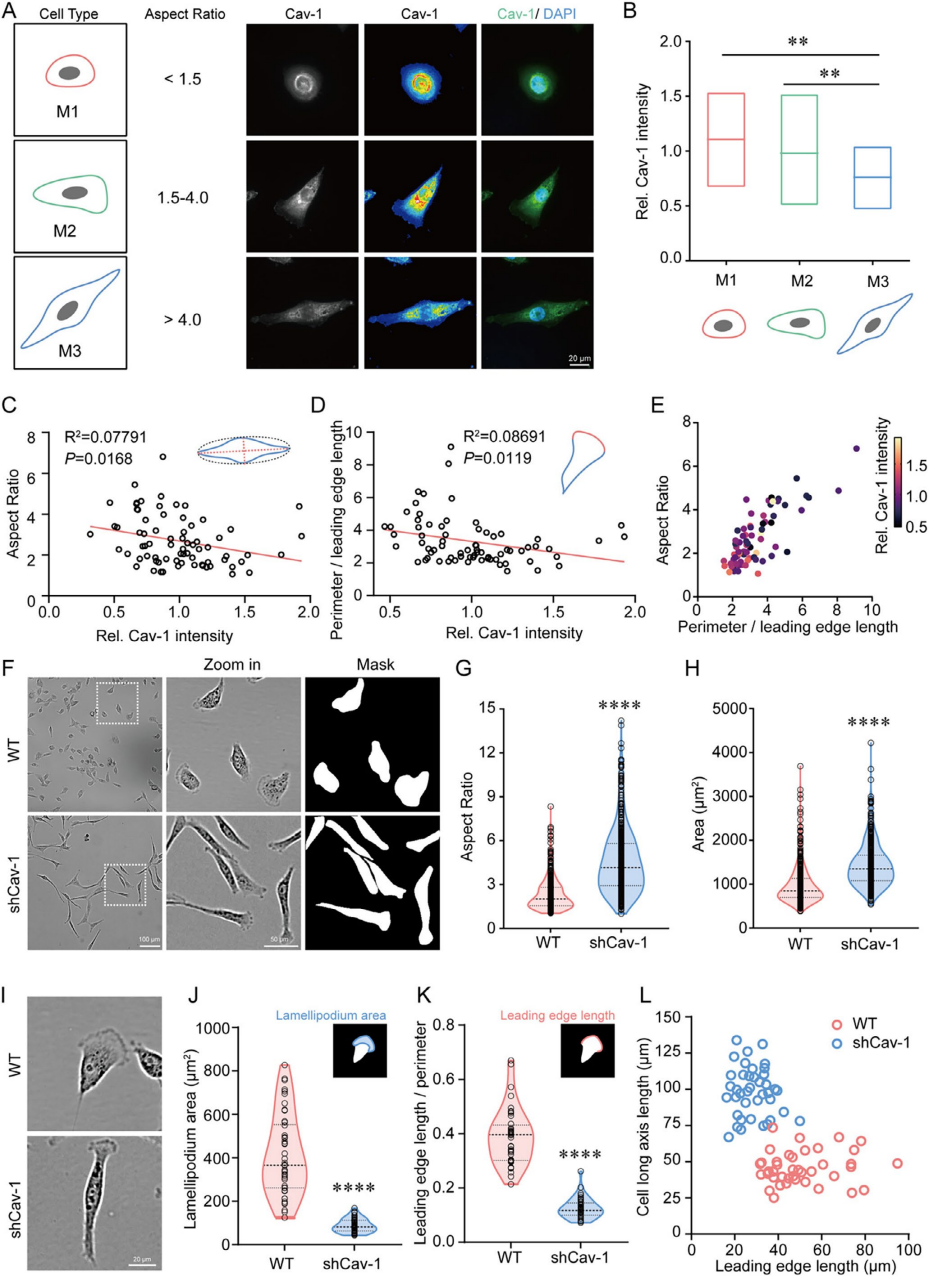
Cav‐1‐dependent cancer cell morphology. (A) Schematic diagram of cell types categorised by aspect ratio (left column) and immunofluorescence images showing Cav‐1 (green) expression in different cell types, with nuclei stained with DAPI (blue in merged images, right column). Scale bar = 20 μm. (B) Quantified Cav‐1 levels in M1 (red), M2 (green) and M3 (blue) cell types. Box and horizontal bars indicate min/max and median values, respectively. *n* = 59, **p < 0.01 (C) Linear regression of aspect ratio and relative expression of Cav‐1. (D) Linear regression of perimeter/leading edge length and relative expression of Cav‐1. (E) Multiple regression of aspect ratio, perimeter/leading edge length and relative expression of Cav‐1. (C–E) *n* = 73. Quantification of the aspect ratio (G) and area (H) of WT and shCav‐1 cells in (F). *n* = 484 in WT group and *n* = 487 in shCav‐1 group, ****p < 0.0001. (I) Representative images of lamellipodium in wild type (WT) and Cav‐1 silencing (shCav‐1) cells. Scale bar = 20 μm. (J,K) Quantification of the lamellipodium area (I) and leading‐edge length/perimeter (J) of WT and shCav‐1 cells in (H). ****p < 0.0001. *n* = 46 in WT group and *n* = 49 in shCav‐1 group, (L) Scatter plot illustrates the variation in cell shape across the dimensions of cell long axis length and leading‐edge length. *n* = 40.

In the previous analysis, after simplifying the complexity in multiple morphological descriptors, aspect ratio and symmetry ratio were selected as two principal parameters to define cancer cell morphology. To facilitate the quantification of symmetry ratio, we introduced the concept of leading‐edge length and subsequently grouped cells into three morphological categories (M1, M2 and M3) according to their aspect ratio (Figure 2A). To investigate whether Cav‐1 is involved in the regulation of tumour cell morphology, we analysed Cav‐1 expression levels in the three cell categories using immunofluorescence. Type M3 cells, which have a higher aspect ratio, showed lower expression of Cav‐1 when compared to M1 and M2 cell types (Figure 2A,B). Further binary regression analysis was performed to quantify the association between Cav‐1 expression and aspect ratio/leading edge length (Figure 2C,D). The results revealed a correlation between cell morphology and Cav‐1 levels in cancer cells. The same correlation was shown by multiple regression analysis (Figure 2E).

To further investigate the association between Cav‐1 expression and the morphology of breast cancer cells, Cav‐1 was knocked down in MDA‐MB‐231 cells using Cav‐1 shRNA (Figure S1A,B). Classification of mixed wild type (WT) and Cav‐1 silencing (shCav‐1) cells according to aspect ratio showed that there were more WT cells in M1 subtype, while there was a higher percentage of shCav‐1 cells in the M3 subtype (Figure S1C). The cellular morphology of MDA‐MB‐231 cells underwent a transition from M1 to M3 upon silencing of Cav‐1 (Figure S1D), resulting in an elongated shape of single cells in the shCav‐1 group (Figure 2F). Statistical analysis revealed that the aspect ratio and cell area were significantly increased in shCav‐1 cells (Figure 2G,H) compared with WT MDA‐MB‐231 cells. The leading‐edge length of shCav‐1 cells decreased, while the cell long axis significantly increased, allowing a clear differentiation between the two cell groups (Figure 2I). In addition, it was observed that the shCav‐1 cells exhibited a significantly reduced area of lamellipodia, along with a decreased ratio of cell leading edge to cell perimeter (accounting for the differences in cell area) (Figure 2J–L). This implies that a decrease in Cav‐1 expression inhibits the generation of lamellipodia. Cav‐1‐dependent morphological heterogeneity with collective polarity was also found in cancer cells of tumour‐bearing mice (Figure S2). Therefore, the expression level of Cav‐1 is correlated with cancer cell morphology, and a decrease in Cav‐1 expression levels may cause changes in cell morphology, resulting in a more elongated phenotype.

### 3.3 Cav‐1 Downregulation Induced Cell Morphological Deformation

**FIGURE 3:**
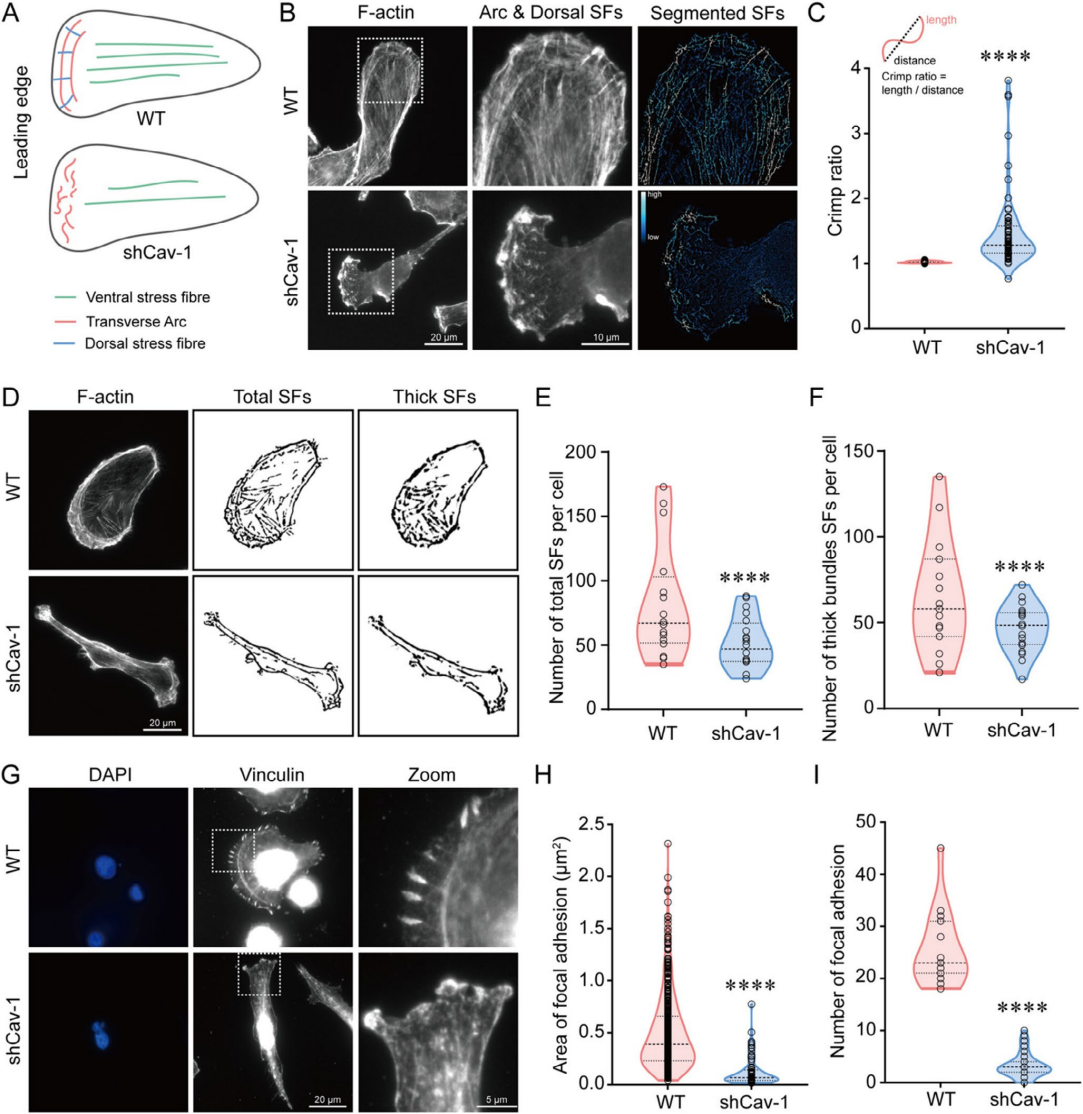
Cav‐1 downregulation induced cell morphological deformation. (A) Schematic representation of stress fibres in WT and shCav‐1 cells. (B) Representative images of global stress fibres (left column), transverse arcs and dorsal stress fibres (middle column) and their segmentations (right column) visualised by phalloidin in WT and shCav‐1 cells. Scale bar = 20 μm (in left column) and Scale bar = 10 μm (in middle column). (C) The crimp ratio was used to quantify the degree of curling of transverse arcs stress fibres in (B), *n* = 52 in WT group and *n* = 72 in shCav‐1 group, ****p < 0.0001. (D) Representative images of actin filaments visualised by phalloidin in WT and shCav‐1 cells (left column). The masks for total stress fibres (middle column) and thick stress fibres (right column) obtained through the ridge detection plugin analysis. Scale bar = 20 μm. (E,F) The average numbers of total (E) and thick filaments (F) of WT and shCav‐1 cells were calculated. (E) *n* = 16 in WT group and *n* = 20 in shCav‐1 group, (F) *n* = 15 in WT group and *n* = 20 in shCav‐1 group, ****p < 0.0001. (G) Representative images of FAs visualised by vinculin antibody staining, respectively, in WT and shCav‐1 cells. Nuclei were stained with DAPI (blue, left column). Scale bar = 20 μm (in cell images) and Scale bar = 5 μm (in the magnified box). Quantifications of FAs area (H) and number (I) of each group in (A). (H) *n* = 477 in WT group and *n* = 72 in shCav‐1 group, (I) *n* = 19 in WT group and *n* = 42 in shCav‐1 group, ****p < 0.0001.

Cell shape is governed by the stress fibres, which generate tractions at the cell‐substrate interface [30]. To investigate the cause of Cav‐1 knockdown induced cancer cell deformation, we first examined whether the decrease of Cav‐1 affected the three types of stress fibres (Figure 3A). The shCav‐1 cells showed weak cytoskeletal structures, especially at the lamellipodial region (Figure 3B). In WT cells, pronounced transverse arcs with straight dorsal stress fibres were observed, while in the shCav‐1 cells, the originally thick transverse arcs disassembled, resulting in shorter and crimpier fibres (Figure 3B,C). In addition, Cav‐1 knockdown also affected the ventral stress fibres. Quantitative analysis of the number of intracellular stress fibres revealed a significant decrease in the total amount of stress fibres, the number of thick bundles and the number of stress fibres in shCav‐1 cells (Figure 3D–F).

FAs serve as points of contact between cells and the ECM, and play a crucial role in maintaining cell morphology on a two‐dimensional plane. Therefore, the expression and distribution of vinculin, a protein in the FA complex, were used to estimate the changes in FAs between WT and shCav‐1 cells. In WT cells, thick FA assemblies were observed at the leading edge of the cell lamellipodia, whereas in shCav‐1 cells there were almost no mature FA signals (Figure 3G). Quantitative analysis showed a significant decrease in both area and number of FAs in shCav‐1 cells (Figure 3H,I). Taken together, reduced Cav‐1 altered the cellular morphology of MDA‐MB‐231 cells by remodelling the cytoskeletal network and FA assembly.

### 3.4 Modelling the Evolution of Cancer Cells Shape

**FIGURE 4:**
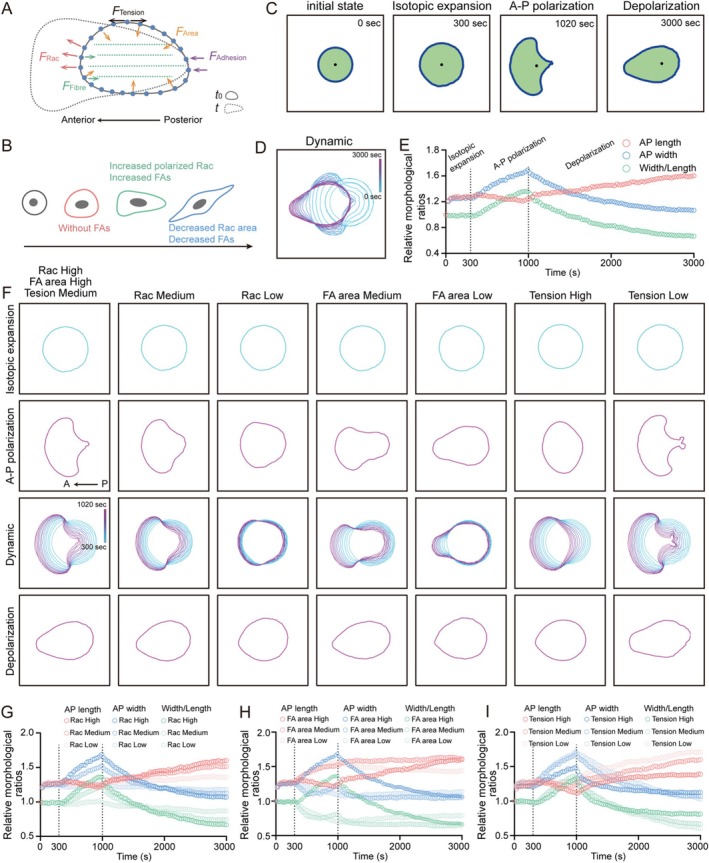
The computational simulation of cancer cell morphological evolution. (A) Representative cartoon to summarise cancer cell morphological change behaviour controlled by Rac1‐mediated protrusive force, cortical tension along the basal membrane, focal adhesions and their linked local stress fibres. (B) Cartoons for the forces change during the steps of the simulation. (C) Representative shapes of cancer cells in one WT during the indicated steps. (D) Boundary outlines mark the temporal changes (colour bars) of one representative cell during the morphological evolution. (E) Single‐factor simulation of dynamic changes of A‐P length, A‐P width and width/length ratio of a typical cancer cell during the whole morphological evolution. (F) Single‐factor simulation of cancer cell morphological evolution with the indicated backgrounds during the A‐P polarisation and depolarization phases. (G) Single‐factor simulation of dynamic changes of A‐P length, A‐P width and width/length ratio of a cancer cell with the inhibition of Rac during the whole morphological evolution. (H) Single‐factor simulation of dynamic changes of A‐P length, A‐P width and width/length ratio of a cancer cell with the inhibition of FAs during the whole morphological evolution. (I) Single‐factor simulation of dynamic changes of A‐P length, A‐P width and width/length ratio of a cancer cell with the regulation of cortical tension during the whole morphological evolution.

To explore the potential principles of morpho‐dynamics, we exploited the computational modelling to theoretically simulate the morphological evolution of cancer cells, with reference to the sequential evolutionary phenotypes and potential regulators of cellular morphology that we observed (Figure 4A,B; see Section 2). Our optimised vertex model represented the morphological evolution of cancer cells by three representative shapes: (1) round‐shaped cell showed lamellipodium with no specific direction of expansion; (2) droplet‐like cell with anterior–posterior (A‐P) polarity; (3) spindle‐like cell with weakened lamellipodium and elongated cell body (Figure 4C and Movie 1). Dynamics of morphological change and the A‐P length/width represented lamellipodium size showed the cellular morphological features during the processes (Figure 4D,E). In the first isotopic expansion step, rounded cells expand isotopically on the substrate without the force generated by stress fibres and FAs. In the second A‐P polarisation step, with the increase of Rac activity, stress fibres and the formation of A‐P cell polarity, the cancer cells shape deformed from round to droplet‐like. In the third depolarization step, reduced Rac and FAs area result in shrinkage of lamellipodium and diminution of A‐P polarity which shaped cell into spindle‐like which is similar to that we observed in shCav‐1 cells. Rac and FAs related morphological changes of the cells from droplet‐like to spindle‐like implied the regulatory role of Cav‐1 in the evolution of cell morphology. In addition, it is important to note that a certain level of movement is a prerequisite for cellular metamorphosis.

Further, we modified key variables individually (strength of Rac, area of FAs or strength of cortical membrane tension) in our simulation, allowing us to decouple the effects of a single mechanism from others. During the round to droplet deformation of cell shape, Rac inhibition strongly blocked the morphological change with smaller lamellipodium area and weaker A‐P polarity (Figure 4F,G). When the area of FAs was reduced, the lamellipodium area at the leading‐edge became smaller in the A‐P polarisation step and elongation was suppressed in the depolarization step (Figure 4F,H). To note, our simulations show that cell membrane tension is a potentially important regulator of cancer cell morphology. Weaker tension accelerates the process of morphological change in cells (Figure 4F–I). Our simulations showed that the balance between protrusion force, FAs and cortical tension determines the direction of changes in cellular morphology, highlighting that Cav‐1‐related remoulding of the cytoskeleton and membrane tension may be critical for the morphological evolution of cancer cells.

### 3.5 Cav‐1‐Related Small GTPases Regulate Cytoskeletal Reorganisation

**FIGURE 5:**
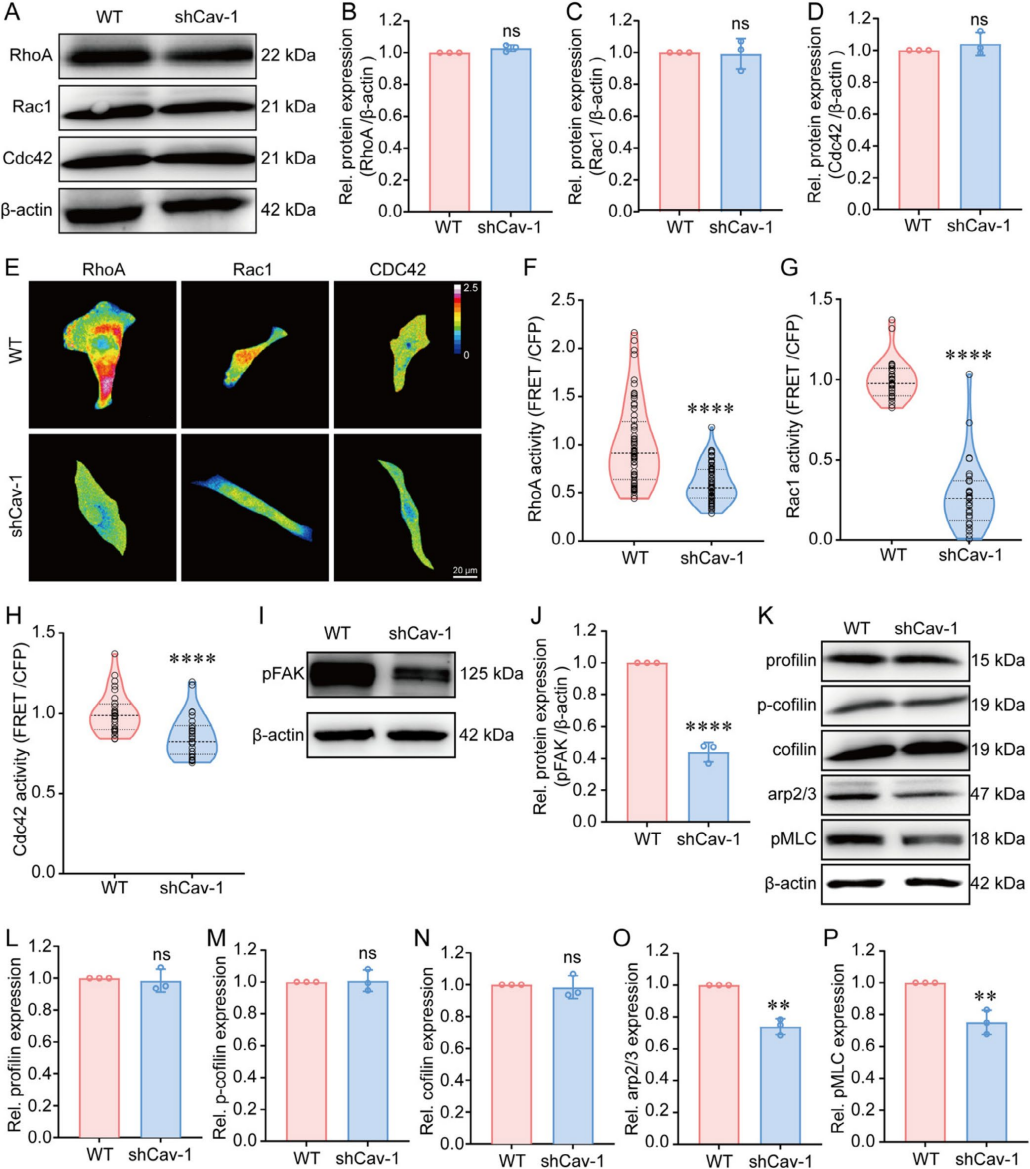
Cav‐1 related small GTPases regulated cytoskeletal reorganisation. (A) Western blot of RhoA, Rac1 and Cdc42 in WT and shCav‐1 cells. β‐actin was used as a loading control. (B–D) Quantification of protein expression levels of RhoA, Rac1 and Cdc42 in WT and shCav‐1 cells, as shown in (A). Data are presented as the mean ± SD. *n* = 3. (E) Representative FRET images of RhoA, Rac1 and Cdc42 activities in WT and shCav‐1 cells. Scale bar = 20 μm. (F–H) Quantification of FRET efficiencies in (E). RhoA, Rac1 and Cdc42 activity was measured as YFP/CYP. (F) *n* = 60, (G) *n* = 31 in WT group and *n* = 29 in shCav‐1 group, (H) *n* = 28 in WT group and *n* = 31 in shCav‐1 group, ****p < 0.0001. (I) Western blot of pFAK in WT and shCav‐1 cells. β‐actin was used as a loading control. (J) Quantification of protein expression levels of pFAK in WT and shCav‐1 cells, as shown in (I). Data are presented as the mean ± SD. *n* = 3, ****p < 0.0001. (K) Western blot of Profilin, p‐cofilin, cofilin, Arp2/3 and pMLC in WT and shCav‐1 cells. β‐actin was used as a loading control. (L–P) Quantification of the protein expression levels of Profilin, p‐cofilin, cofilin, Arp2/3 and pMLC in WT and shCav‐1 cells, as shown in (K). Data are presented as the mean ± SD. *n* = 3, **p < 0.01.

The Rho family of small guanosine triphosphatases (GTPases) plays a key regulatory role in the organisation of the actin cytoskeleton through the distinctive effects of its family members [31, 32]. For example, the activation of Rho, Rac and Cdc42 has effects on the formation of stress fibres, construction of lamellipodia and filopodia, respectively [33]. To determine whether Cav‐1 is necessary for cytoskeletal reorganisation through the Rho GTPases, we first investigated the effect of Cav‐1 downregulation on the expression levels of RhoA, Rac1 and Cdc42. The total protein levels of RhoA, Rac1 and Cdc42 showed no significant differences between WT and shCav‐1 cells, indicating that the downregulation of Cav‐1 does not affect the expression of these small GTPases (Figure 5A–D). Considering the role of Rho GTPases as molecular switches, we used FRET technology to measure their activation. The FRET analysis revealed a significant decrease in the activation levels of RhoA, Rac1 and Cdc42 in shCav‐1 cells (Figure 5E–H). In particular, the lack of Rac activity at the leading edge of the cell also explains the absence of large lamellipodia in shCav‐1 cells. These results suggest that the downregulation of Cav‐1 may modulate cytoskeletal rearrangement by reducing the activation of small GTPases.

To further elucidate the mechanism by which Cav‐1 regulates cellular cytoskeletal dynamics through the Rho GTPase family, we investigated the upstream and downstream signals involved in this Cav‐1 dependent cellular morphology. FA kinase (FAK) has the ability to regulate the activity of Rho family GTPases (RhoA, Rac and Cdc42) through direct interaction and phosphorylation of GTPase‐activating proteins (GAPs) or guanine nucleotide‐exchange factors (GEFs), which can lead to local actin assembly to form stress fibres, lamellipodia or filopodia [34]. There was a significant decrease in phosphorylated FAK (p‐FAK) in shCav‐1 cells, suggesting that downregulated Cav‐1 reorganised the actin cytoskeleton through the FAK‐Rho GTPase signalling (Figure 5I,J). Regarding the downstream effectors of Rho GTPases, the downregulation of Cav‐1 specifically decreased the expression of Arp2/3 and phosphorylated myosin light chain (p‐MLC) (Figure 5K–P), suggesting that Cav‐1 depletion may primarily inhibit the Rac1 signalling and lamellipodia formation, consequently affecting cell morphology. In conclusion, decreased Cav‐1 affects the organisation of the actin cytoskeleton through the FAK‐regulated Rho GTPases signalling, thereby influencing the formation of FAs, stress fibres and lamellipodia, leading to changes in cellular morphology.

### 3.6 Cav‐1‐Dependent Self‐Renewal Capability

**FIGURE 6:**
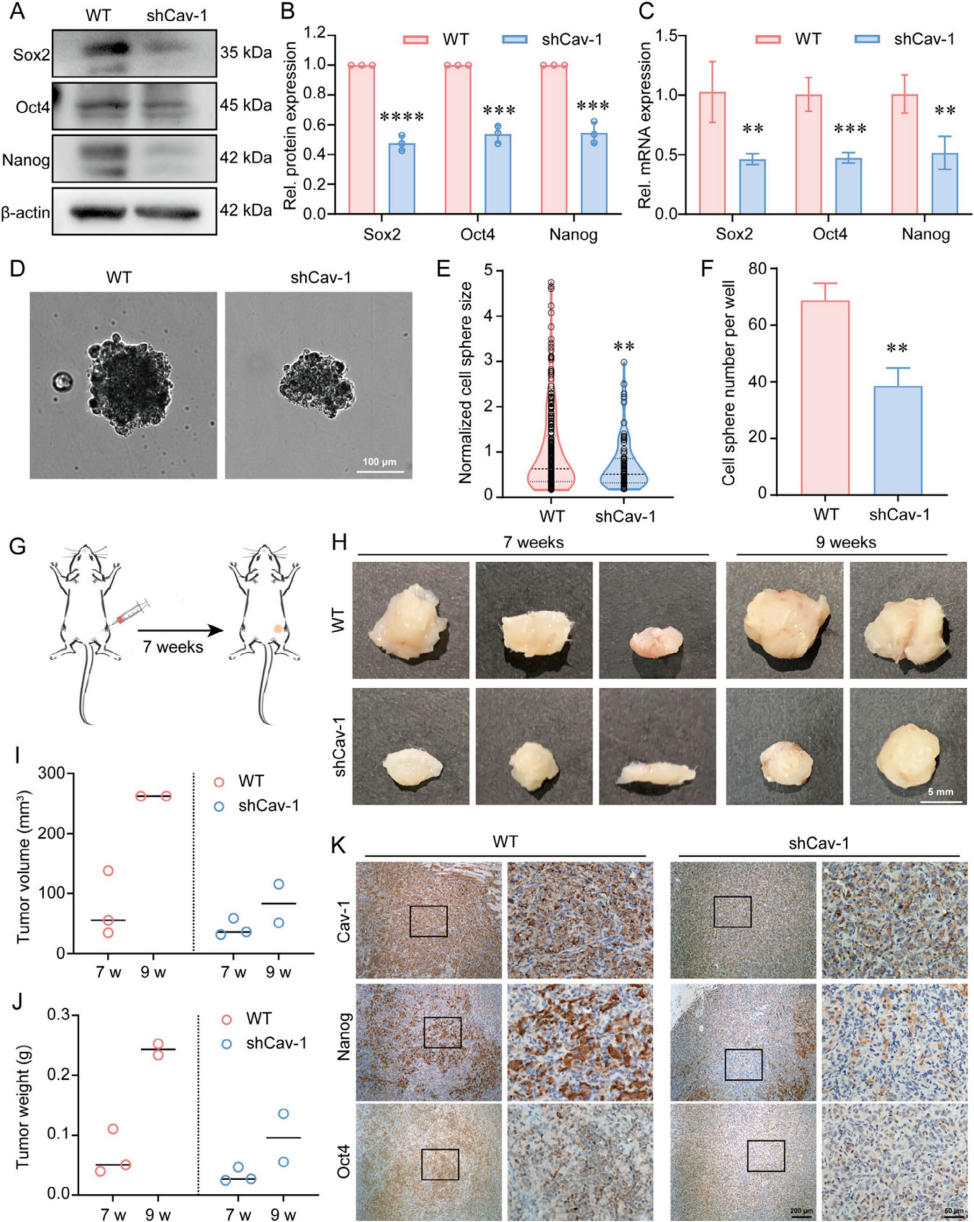
Cav‐1 dependent self‐renewal capability. (A) Western blot of Nanog, Sox2 and Oct4 in WT and shCav‐1 cells. β‐actin was used as a loading control. (B) Quantification of the protein expression levels of Nanog, Sox2 and Oct4 in WT and shCav‐1 cells. Data are presented as the mean ± SD. *n* = 3, ***p < 0.0005, ****p < 0.0001. (C) Quantification of mRNA levels of Nanog, Sox2 and Oct4 in WT and shCav‐1 cells. (D) Bright field images of WT and shCav‐1 cell spheres. Scale bar = 100 μm. Quantifications of cell spheres size (E) and number (F) of WT and shCav‐1 cell spheres in (A). (E) *n* = 273 in WT group and *n* = 83 in shCav‐1 group, **p < 0.01. (G) Schematic representation of the mouse tumour model. (H) MDA‐MB‐231 and shCav‐1 cells (5 × 10^6^ cells in 100 μL of Matrigel) were respectively injected subcutaneously into NCG mice. Mice were divided into two groups (five mice/group). Each group consisted of three mice sacrificed at the 7th week and 2 mice sacrificed at the 9th week, with tumours extracted from each of them. Scale bar = 5 mm. Tumour volume (I) and tumour weight (J) of tumour bearing mice. (K) Immunohistochemical staining of Cav‐1, Nanog and Oct4 in WT and shCav‐1 tumour sections. Scale bar = 200 μm. Data are normalised to WT control. *n* = 4, **p < 0.01, ***p < 0.0005.

The self‐renewal capacity of tumour cells is closely associated with cancer malignancy, including recurrence, metastasis and fast growth despite therapy. To investigate the role of Cav‐1 in regulating self‐renewal capacity in MDA‐MB‐231 cells, the expression and distribution of typical self‐renewal regulators (Sox2, Oct4 and Nanog) were examined using immunofluorescence staining. It was observed that the expression of Sox2 (Figure S3A), Oct4 (Figure S3D) and Nanog (Figure S3G) was significantly reduced in shCav‐1 cells. Since their functions as transcription factors, both the overall expression levels and nuclear/cytoplasmic ratio were assessed. The expression levels and nuclear localization of Oct4 and Nanog were decreased in shCav‐1 cells (Figure S3D–I), while no significant difference was observed in the nuclear‐to‐cytoplasmic ratio of Sox2 between the two cell groups (Figure S3A–C). Further Western blot analysis revealed a significant decrease in the protein levels of these three self‐renewal regulators upon Cav‐1 knockdown (Figure 6A,B). In addition, real‐time qPCR experiments revealed a notable reduction in the mRNA levels of Sox2, Oct4 and Nanog in shCav‐1 cells (Figure 6C). Thus, the downregulation of Cav‐1 inhibited both the expression and nuclear translocation of self‐renewal transcription factors, thereby impairing the self‐renewal capacity of the cells.

To further confirm the inhibitory effect of decreased Cav‐1 levels on the self‐renewal capacity of cancer cells, tumour sphere formation assays and xenograft tumour mouse models were conducted. It was observed that knockdown of Cav‐1 resulted in a significant decrease in the size and number of MDA‐MB‐231 cell spheres (Figure 6D–F). To construct the tumour xenograft model, WT and shCav‐1 MDA‐MB‐231 cells were injected subcutaneously into female NCG severe immunodeficient mice (5 weeks old), followed by tumour tissue collection at Weeks 7 and 9 post‐inoculation, allowing us to observe tumour size, measure tumour weight and calculate tumour volume (Figure 6G). Tumours generated by shCav‐1 cells were generally smaller than those generated by WT cells (Figure 6H–J). Although tumour tissues harvested at 9 weeks showed some growth, this did not alter the overall trend. The tumour tissues were fixed and sectioned for immunohistochemical staining of Cav‐1, Nanog and Oct4. It was observed that the expression of Nanog and Oct4 was more robust in WT cells compared to shCav‐1 cells, consistent with the results on cells in vitro (Figure 6K). In conclusion, the self‐renewal capacity of MDA‐MB‐231 breast cancer cells is dependent on Cav‐1 and Cav‐1 downregulation directly affects the transcription of Sox2, Oct4 and Nanog.

### 3.7 Spreading Area Does Not Affect the Self‐Renewal of Tumour Cells

We used micropattern printing technology to confine the cell edge and ensure that both WT and shCav‐1 cells had the same shape and spreading area of 1000 μm^2^ (Figure S4A). We then examined the expression of self‐renewal markers in WT and shCav‐1 MDA‐MB‐231 cells on micropatterns. Interestingly, we observed no significant differences in the expression of Oct4 and Sox2 between the WT and shCav‐1 cell groups (Figure S4B,E). Furthermore, the intensity (Figure S4C,F) and nuclear localization of these three transcription factors were similar in both groups (Figure S4D–G). These results suggest that the effect of Cav‐1 on cell self‐renewal capacity is achieved through changes in cell morphology, rather than cell spreading and area. After controlling for cell morphology, downregulation of Cav‐1 expression does not inhibit the expression and nuclear localization of these self‐renewal markers.

It is still unclear how cells combine morphological adjustments and genetic phenotypic remodelling, but the correlation between changes in global cell morphology and nuclear structure may be one of the potential strategies. After cell shape changes, the nucleus can undergo significant structural changes due to compression and stretching by the cytoskeleton or cell membrane, affecting chromatin accessibility and gene expression. Here, we found that spindle‐shaped shCav‐1 cells showed a reconstructed nuclear morphology (Figure S5A) with decreased nuclear area, increased aspect ratio, height and volume (Figure S5B–G). Contrasting the changes in cell morphology and actin cytoskeleton (Figure 3D) between WT and shCav‐1 cells, the deformation of the nucleus in shCav‐1 cells is probably a result of the pressure from the cell membrane and cytoskeletal fibres on the non‐polar sides of the cells. This can further explain that the silence or downregulation of Cav‐1 induced Rac and Rho dependent actin cytoskeleton remoulding resulted cellular morphology is an important regulator of gene expression and behavioural shifts of cancer cells, thus emphasising that the morphological evolution of cancer cells is an important driver of their gene expression and malignant phenotypic transformation. Thus, although we do not have definitive evidence, it is predictable that the morpho‐mechanical regulation of the nucleus may bridge the gap between cell morphological diversity and cell phenotype.

## 4 Discussion

**FIGURE 7:**
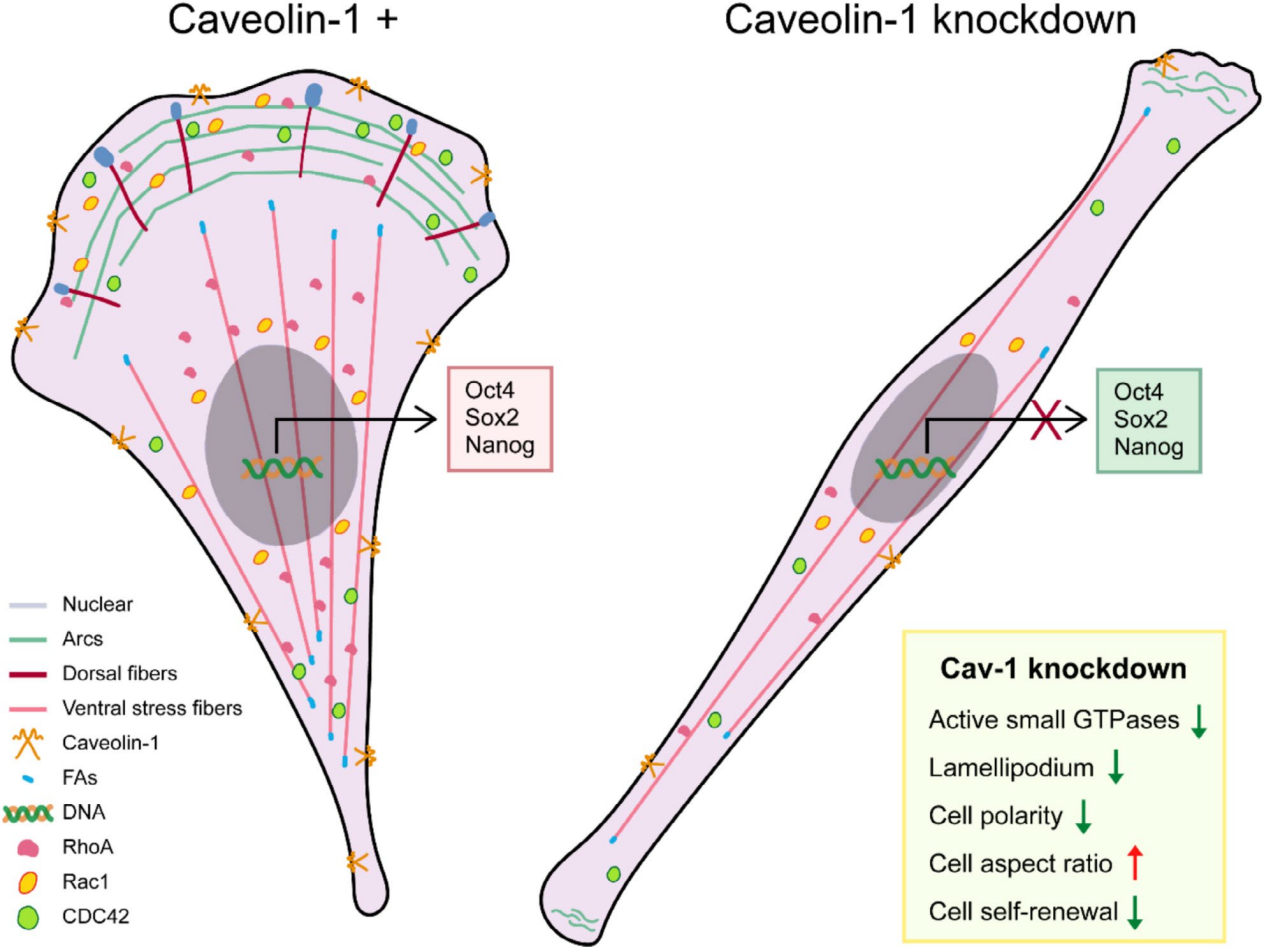
Cav‐1‐dependent cancer cell morphological and phenotype changes. In the MDA‐MB‐231 triple‐negative breast cancer cell line, Cav‐1 can induce changes in cell morphology, leading to nuclear deformation, thereby impacting the cell's self‐renewal capability. During this process, Cav‐1 can influence the activity and distribution of three small GTPases through the FAK‐Rho signalling pathway. Cells lacking Cav‐1 exhibit decreased activity of small GTPases, resulting in reduced lamellipodia formation, reshaping of the cytoskeleton, loss of cell polarity, decreased focal adhesion assembly and alteration in cell morphology, which in turn affects nuclear shape and may influence gene expression, including that of self‐renewal‐related transcription factors.

In this study, we had segmented and described the 2D morphological features of single cancer cells and clustered the breast cancer cells into three classes with basic morphological descriptors. These three types of single‐cell morphological features remind us that the morphological diversity of cancer cells might be a stepwise evolutionary process. Using single gene silencing of Cav‐1, we reproduce the morphological evolution of cancer cells from round, droplet to spindle‐like. We found that the attenuation of dorsal stress fibres, the assembly of FAs and the disorder of transverse arc fibres are responsible for the morphological transformation of the shCav‐1 breast cancer MDA‐MB‐231 cells (Figure 7). In addition, our modified vertex model theoretically reproduced the evolutionary process of cellular morphology. Small GTPases and FAK signalling have been implicated in Cav‐1 knockdown induced cytoskeletal remodelling and FA assembly. Furthermore, both in vitro and in vivo studies have demonstrated that Cav‐1 dependent morphological changes are closely associated with the self‐renewal capacity of breast cancer cells.

The shape of a cell is not static, especially for moving cells, but a spatiotemporal dynamic sequence of cell geometry. Thus, the collection of multicellular morphology that we observe at one point in time can globally characterise the shape of a cell type. However, the mechanism by which global morphology is generated and maintained at the cellular scale is not understood. Some morphologically simple and stereotyped cells with fine association with the dynamics of structural molecules are favourable models for studying cellular shape determination. For example, the morphological dynamics of the global shape of keratocytes could be described by a model of an actin network in a flexible bag [35]. In this study, to explore the general morphological diversity of cancer cells, after extracting single‐cell morphological feature descriptors of MDA‐MB‐231 cells, the cells were categorised into three distinct clusters by the *k*‐means clustering algorithm. The knockdown of Cav‐1 launched the morphological evolution of cancer cells from round, droplet to spindle‐like. Indeed, the diversity of cellular morphologies displayed in our cancer cell morphology spectrum appears to be similar to the multiple intermediate cell states between the epithelial and the mesenchymal states generated by spontaneous cancer cell EMT during tumorigenesis. These intermediate state cells may have stronger stem cell properties, making them more likely to survive after anticancer therapies and spread to distal regions to form metastatic colonies [14].

The actomyosin network, cell membrane and cell matrix adhesion largely determine the morphology of motile cells in real time. Uniform and isotropic contraction shapes cells to be round or round‐like, whereas during migration, spatially dynamic contractile forces create A‐P polarity and make posterior retractions [36]. In this study, changes in the cytoskeletal network and FAs during this process further explain the potential mechanisms of morphological diversity of cancer cells. In breast cancer MDA‐MB‐231 cells, the lamellipodium assembled by transverse arcs and dorsal stress fibres provides the cell with A‐P morphological polarity. Interestingly, while inhibition of Cav‐1 reduces dorsal stress fibres and tension, transverse arcs show a curly‐like appearance, unable to support extensive lamellipodium, resulting in a spindle‐like shape of shCav‐1 cells. The lamellipodium at the front of the cell, which lacks contractility, struggles to form mature FAs. Thus, Cav‐1 inhibited cells morphologically lose the extensive lamellipodium at the leading edge and global front‐rear polarity. We further analysed the potential mechanisms by which Cav‐1 is involved in the regulation of cytoskeletal networks. Knockdown of Cav‐1 causes changes in the distribution and activity of the small GTPase and given the changes in the downstream molecules of Arp2/3 and pMLC, we suggest that Rac and Rho, which are involved in the regulation of the lamellipodium and ventral stress fibre, are essential for cell shape remodelling. The interaction and feedback between the actomyosin network and Cav‐1 may be involved in the regulation of cell morphology and motility in several ways. On the one hand, Cav‐1 could directly interact with activated Rac1 and Cdc42 to regulate FA kinase activity and the actin stress fibre network, resulting in senescence‐associated morphological deformations in the senescent cells [37]. On the other hand, intercellular Cav‐1 vesicles have been observed to make retrograde flow along the transverse arcs towards the cell centre [38] and the dynamics of caveolae are largely associated with the intercellular contraction and actin networks. In addition, although we focused on the effect of Cav‐1‐dependent cytoskeletal regulation on cell morphology, membrane tension is also a key factor in regulating cell morphology due to its ability to coordinate cell protrusion and retraction. The physical properties of the cell membrane depend on its lipid components and microstructures. As a cell membrane reservoir, caveolae are able to dynamic regulation of membrane tension. Through the flattening and breakdown of caveolae, cells are able to quickly dampen the sudden spikes in membrane tension triggered by mechanical stress [39]. Consequently, we hypothesise that Cav‐1 deficiency is highly likely to participate in the modulation of membrane mechanical properties by altering its physical characteristics. Further research will be necessary to determine how membrane tension contributes to cell shape and motility.

Self‐renewal capacity is a fundamental requirement for cancer cells to be able to continue to evolve spontaneously and is a crucial engine for driving tumour progression [40]. As a key feature of cancer stem cells (CSCs), the ability of self‐renewal could maintain stem cell populations and accumulate mutations. Therefore, targeted therapy of CSC self‐renewal is considered a potential clinically relevant anti‐cancer strategy [41, 42]. Here, after confirming the regulatory role of Cav‐1 in breast cancer cell morphology, we investigated the inhibitory effect of Cav‐1 silencing on cellular self‐renewal capacity both in vitro and in vivo. Silence of Cav‐1 significantly reduced the expression and nuclear transport of self‐renewal‐related transcription factors, such as Sox2, Oct4 and Nanog. Additionally, it decreased the size of multicellular spheroids and tumours in tumour‐bearing mice.

Cell shape, as a global feature, allows a cell to translate its local shape features into intracellular signals and integrate them into cell physiology [43]. Cell shape of single cells in developing embryos and differentiated tissues is usually associated with specific functions, and changes in the morphology of tumour cells imply transformation in their phenotypes and functions [8]. How cell morphology affects its behaviour and physiological function remains an open question. A number of microscale technologies, including micropatterns, microchannels and microwells, allow precise control of cell morphology to study the regulation of cell morphology. Here, micro‐patterns were used to confine the single cell spreading area, showing that Cav‐1 dependent cell morphology, but not the cell spreading size, influences the self‐renewal capability of breast cancer cells. However, compared with 2D cultured cells, 3D cultured cells are more similar to cells growing in vivo in terms of morphology, gene expression, signal transduction and metabolism. The cytoskeletal network formed in a 3D environment confers upon the cells morphological differences from those of cells growing on a 2D substrate. Although the morphological characteristics of cells cultured in 2D and 3D environments are not entirely compatible, there are still some similar overall features, such as polarity along the long axis. Therefore, the morphological characteristics of cells in 3D environments need to be further evaluated using a co‐culture strategy that may bridge the shape features of 2D and 3D tumour cells [44]. Since the cell nucleus is where the gene undergoes transcription, changes in its structure can affect the transcription process in various ways. Mechanical confinements to control single‐cell geometry revealed that self‐renewal capacity can be modulated by cell morphology. This cell morphology‐associated regulation of self‐renewal capacity may result from the remodelling of nuclear structure and gene expression [45]. For example, deformation of the cell nucleus caused by microfluidic channel confinement can promote fibroblasts to reprogram into neurons through the decrease of histone methylation and DNA methylation [46]. To fully answer this question, further studies are needed to decouple the deformation of the nucleus from the cell body and how mechanical cues affect nuclear mechanics, genome organisation and transcription.

It should be noted that, while our study emphasises how Cav‐1 regulates the static morphology and self‐renewal of breast cancer cells, it is important to recognise that cell shape is inherently dynamic and intimately interconnected with cell movement. Migrating cancer cells continuously remodel their cytoskeleton and adhesion sites, resulting in transient morphologies that enable navigation through complex microenvironments—a process fundamental to metastasis and progression [3, 47]. These shape changes, such as the transition from round to spindle‐like forms observed during Cav‐1 knockdown, mirror the morphological plasticity seen in EMT, which enhances motility and invasive capacity [14]. As cells migrate, mechanical and biochemical signals generated by movement can also impact nuclear deformation and gene expression, feeding back to further influence phenotype [45, 48]. Our computational modelling and experimental results support the idea that cytoskeletal and adhesive dynamics associated with motility are primary drivers of morphological evolution in cancer cells. Thus, targeting regulators that couple cell movement and morphology, such as Cav‐1‐mediated pathways, holds promise for limiting cancer cell plasticity and malignancy.

In summary, the findings presented in this study have revealed the critical role of single‐cell morphology in regulating the self‐renewal capacity of breast cancer cells (Figure 7). After classifying cell morphology into three categories by simple downscaling, we used Cav‐1 silencing and a mathematical vertex model to reproduce the morphological evolution of cancer cells. The attenuation of dorsal stress fibres, the assembly of FAs and the disorder of transverse arc fibres are considered to be the main tools for the morphological evolution of cancer cells. Together, we have unveiled the association between morphological diversity and the biological properties of cancer cells from a morphological evolutionary perspective, suggesting that Cav‐1‐dependent cellular morphological characteristics are critical regulators of the malignancy properties of cancer cells, such as self‐renewal ability.

## Author Contributions

**Shun Li:** conceptualization, data curation, investigation, methodology, funding acquisition, supervision, writing – original draft, writing – review and editing. **Hongyun Duan:** formal analysis, investigation, visualisation. **Lu Yang:** formal analysis, investigation, methodology. **Lingyi Jiang:** formal analysis, investigation, methodology. **Haocheng Bian:** formal analysis, methodology. **Yuqin Jiang:** data curation, formal analysis, investigation. **Yixi Zhang:** data curation, formal analysis, investigation. **Wei Yan:** data curation, formal analysis, investigation. **Qin Yang:** data curation, investigation. **Tingting Li:** conceptualization, funding acquisition, supervision. **Xiang Qin:** conceptualization, funding acquisition, methodology. **Zong‐Yuan Liu:** conceptualization, data curation, investigation, methodology, writing – original draft. **Ningwei Sun:** conceptualization, data curation, investigation, methodology, writing – original draft. **Kai‐fu Yang:** conceptualization, methodology, software, supervision, writing – original draft. **Yiyao Liu:** conceptualization, funding acquisition, supervision, writing – original draft, writing – review and editing.

## Ethics Statement

This study was approved by the Ethics and Research Committees of the University of Electronic Science and Technology of China. The animal work was approved by the Ethics Committee for Animal Experiments of the University of Electronic Science and Technology of China.

## Conflicts of Interest

The authors declare no conflicts of interest.

## Supporting information


**Movie 1.** The computational simulation of cancer cell morphological evolution. Time‐lapse sequence of cancer cell morphological expansion which includes three steps: isotopic expansion, A‐P polarisation and depolarization at the indicated different backgrounds. Green colour marks the cell boundary at basal domain. The unit of time is seconds.

**Figure S1:** Western blot analysis (A) and quantifications (B) of Cav‐1 in WT and shCav‐1 cells. β‐actin was used as a loading control. Data are presented as the mean ± SD, *n* = 3, ****p < 0.0001. (C) The proportion of WT and shCav‐1 cells in the M1, M2 and M3 types of cells. (D) The proportion of M1, M2 and M3 phenotypes in the WT and shCav‐1 cells. (C and D) *n* = 971.
**Figure S2:** Representative H&E staining images WT and shCav‐1 tumour sections from mice. Scale bar = 200 μm.
**Figure S3:** Immunofluorescence images showing Nanog (A), Sox2 (D) and Oct4 (G) (green) in WT and shCav‐1 cells, with nuclei stained with DAPI (magenta in merged images; right column). Scale bar = 20 μm. Violin plot of quantified Nanog (B), Sox2 (E) and Oct4 (H) levels from images (A/D/G), respectively. ****p < 0.0001. Nanog (C), Sox2 (F) and Oct4 (I) distribution from (A/D/G) were represented as the ratio of nuclear‐to‐cytosolic intensities. ****p < 0.0001. (B, C) *n* = 72 in WT group and *n* = 60 in shCav‐1 group, (E, F) *n* = 75 in WT group and *n* = 72 in shCav‐1 group, (H, I) *n* = 64 in WT group and *n* = 70 in shCav‐1 group.
**Figure S4:** Bright field of the PDMS mould (left), green fluorescence channel picture of the culture dish after micropattern printing (middle) and schematic diagram of cells seeded on the micropattern (right). Scale bar = 100 μm. Immunofluorescence images showing Oct4 (B) and Sox2 (E) (yellow) in WT and shCav‐1 cells seeded on micropatterns, with nuclei stained with DAPI (blue in merged images; right column). Scale bar = 30 μm. Violin plot of quantified Oct4 (C) and Sox2 (F) levels from images (B/E), respectively. Oct4 (D) and Sox2 (G) distribution from (B/E) were represented as the ratio of nuclear‐to‐cytosolic intensities. (C, D) *n* = 39 in WT group and *n* = 38 in shCav‐1 group, (F, G) *n* = 79 in WT group and *n* = 70 in shCav‐1 group
**Figure S5:** (A) Images of nuclei in WT and shCav‐1 cells stained with DAPI (blue, left column) along with their corresponding nuclear mask images (right column) in the *x*–*y*, *x*–*z* and *y*–*z* planes. Quantification of the nucleus cross‐sectional area (B), nucleus aspect ratio (C), nucleus height (D), nucleus volume (E) and nucleus convexity (F) of WT and shCav‐1 cells in (A), **p < 0.005, ****p < 0.0001. (B, C) *n* = 41 in WT group and *n* = 42 in shCav‐1 group, (D) *n* = 26 in WT group and *n* = 33 in shCav‐1 group, (E) *n* = 31 in WT group and *n* = 25 in shCav‐1 group, (F) *n* = 28. (G) Schematic depiction of the cellular and nuclear shapes of WT and shCav‐1 cells.

## Data Availability

The data that support the findings of this study are available from the corresponding author upon reasonable request.
