# Supplementary material for: Decoding the Role of Caveolin‐1 in Morphological Diversity and Self‐Renewal of Breast Cancer Cells
Source: Cell Prolif. 2025 Oct 30;59(5):e70137. doi: 10.1111/cpr.70137 (PMC13114796; doi:10.1111/cpr.70137)
Supplement: Supplementary file 2 — Figure S1: Western blot analysis (A) and quantifications (B) of Cav‐1 in WT and shCav‐1 cells. β‐actin was used as a loading control. Data are presented as the mean ± SD, n = 3, ****p < 0.0001. (C) The proportion of WT and shCav‐1 cells in the M1, M2 and M3 types of cells. (D) The proportion of M1, M2 and M3 phenotypes in the WT and shCav‐1 cells. (C and D) n = 971. Figure S2: Representative H&E staining images WT and shCav‐1 tumour sections from mice. Scale bar = 200 μm. Figure S3: Immunofluorescence images showing Nanog (A), Sox2 (D) and Oct4 (G) (green) in WT and shCav‐1 cells, with nuclei stained with DAPI (magenta in merged images; right column). Scale bar = 20 μm. Violin plot of quantified Nanog (B), Sox2 (E) and Oct4 (H) levels from images (A/D/G), respectively. ****p < 0.0001. Nanog (C), Sox2 (F) and Oct4 (I) distribution from (A/D/G) were represented as the ratio of nuclear‐to‐cytosolic intensities. ****p < 0.0001. (B, C) n = 72 in WT group and n = 60 in shCav‐1 group, (E, F) n = 75 in WT group and n = 72 in shCav‐1 group, (H, I) n = 64 in WT group and n = 70 in shCav‐1 group. Figure S4: Bright field of the PDMS mould (left), green fluorescence channel picture of the culture dish after micropattern printing (middle) and schematic diagram of cells seeded on the micropattern (right). Scale bar = 100 μm. Immunofluorescence images showing Oct4 (B) and Sox2 (E) (yellow) in WT and shCav‐1 cells seeded on micropatterns, with nuclei stained with DAPI (blue in merged images; right column). Scale bar = 30 μm. Violin plot of quantified Oct4 (C) and Sox2 (F) levels from images (B/E), respectively. Oct4 (D) and Sox2 (G) distribution from (B/E) were represented as the ratio of nuclear‐to‐cytosolic intensities. (C, D) n = 39 in WT group and n = 38 in shCav‐1 group, (F, G) n = 79 in WT group and n = 70 in shCav‐1 group Figure S5: (A) Images of nuclei in WT and shCav‐1 cells stained with DAPI (blue, left column) along with their corresponding nuclear mask images (right colu [file CPR-59-e70137-s001.docx]

**Supplementary Information**

**Decoding the Role of Caveolin-1 in Morphological Diversity and Self-Renewal of Breast Cancer Cells**

Shun Li ^1^, Hongyun Duan ^2^, Lu Yang ^1^, Lingyi Jiang ^1^, Haocheng Bian ^1^, Yuqin Jiang ^1^, Yixi Zhang ^1^, Wei Yan ^1, 4^, Qin Yang ^1^, Tingting Li ^1^, Xiang Qin, Zong-Yuan Liu ^3 *^, Ningwei Sun ^1 *^, Kai-fu Yang ^2 *^, Yiyao Liu ^1, 4, 5 *^

*^1^ Department of Oncology & Cancer Institute, Sichuan Academy of Medical Sciences, Sichuan Provincial People’s Hospital, and School of Life Science and Technology, University of Electronic Science and Technology of China, Chengdu 610054, Sichuan, P. R. China*

*^2^ MOE Key Laboratory for Neuroinformation, School of Life Science and Technology, University of Electronic Science and Technology of China, Chengdu 610054, Sichuan, P. R. China.*

*^3^ Department of Engineering Mechanics, Institute of Biomechanics and Medical Engineering, Tsinghua University, Beijing 100084, P.R. China.*

*^4^ Traditional Chinese Medicine (TCM) Regulating Metabolic Diseases Key Laboratory of Sichuan Province, Hospital of Chengdu University of Traditional Chinese Medicine, Chengdu 610072, Sichuan, P. R. China*

*^5^ Department of Urology, Deyang People's Hospital, Deyang 618099, Sichuan, P. R. China*

***Corresponding authors:**

Email addresses: Email addresses: zongyu@umich.edu (ZY. Liu), ningwei.sun@univ-tlse3.fr (NW. S), yangkf@uestc.edu.cn (K. Yang), liuyiyao@uestc.edu.cn (Y. Liu)


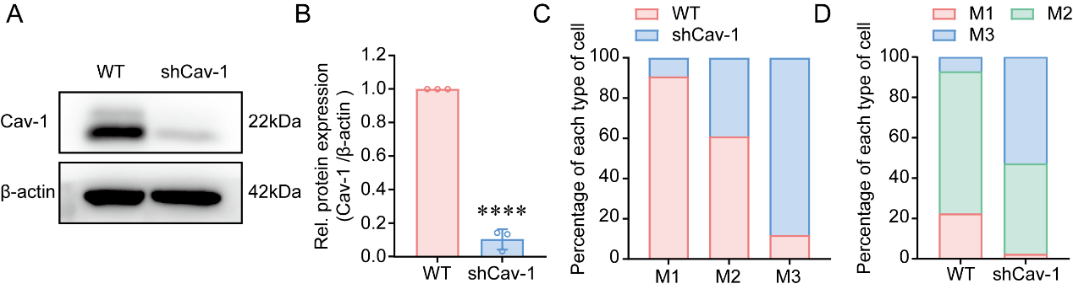


**Figure S1**. (**A**-**B**) Western blot analysis (A) and quantifications (B) of Cav-1 in WT and shCav-1 cells. β-actin was used as a loading control. Data are presented as the mean ± SD, n=3, **** *p*<0.0001. (**C**) The proportion of WT and shCav-1 cells in the M1, M2, and M3 types of cells. (**D**) The proportion of M1, M2, and M3 phenotypes in the WT and shCav-1 cells. (C and D) n = 971.


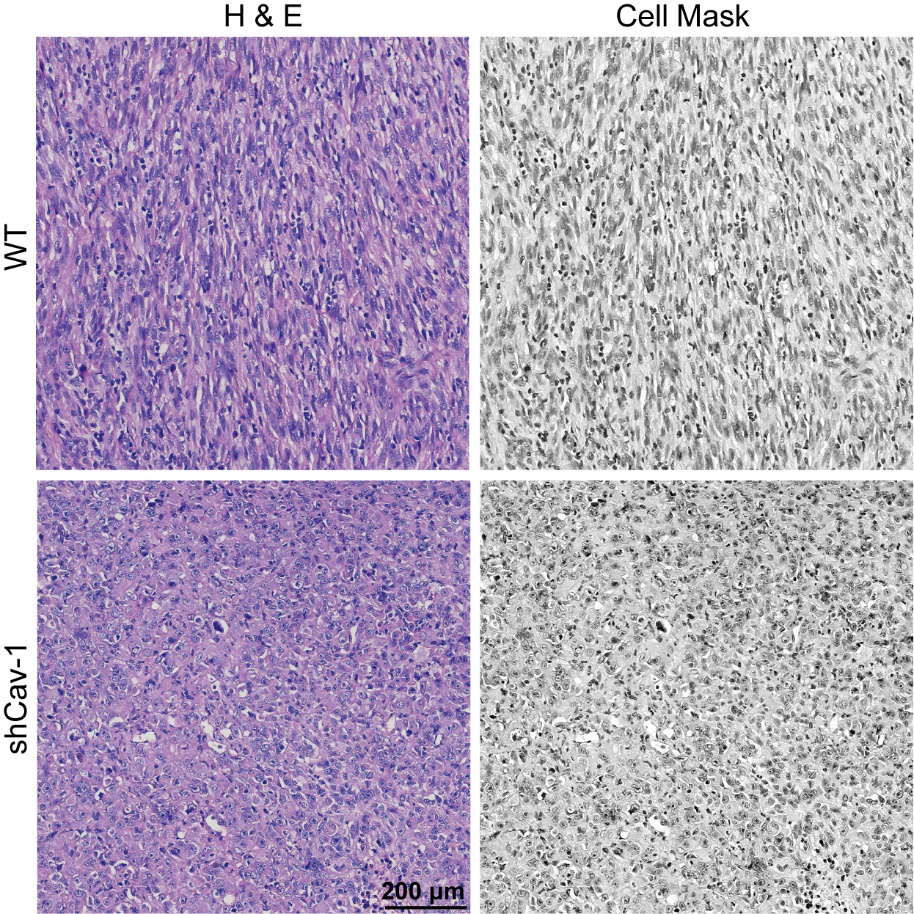


**Figure S2**. Representative H&E staining images WT and shCav-1 tumor sections from mice. Scale bar = 200 μm.


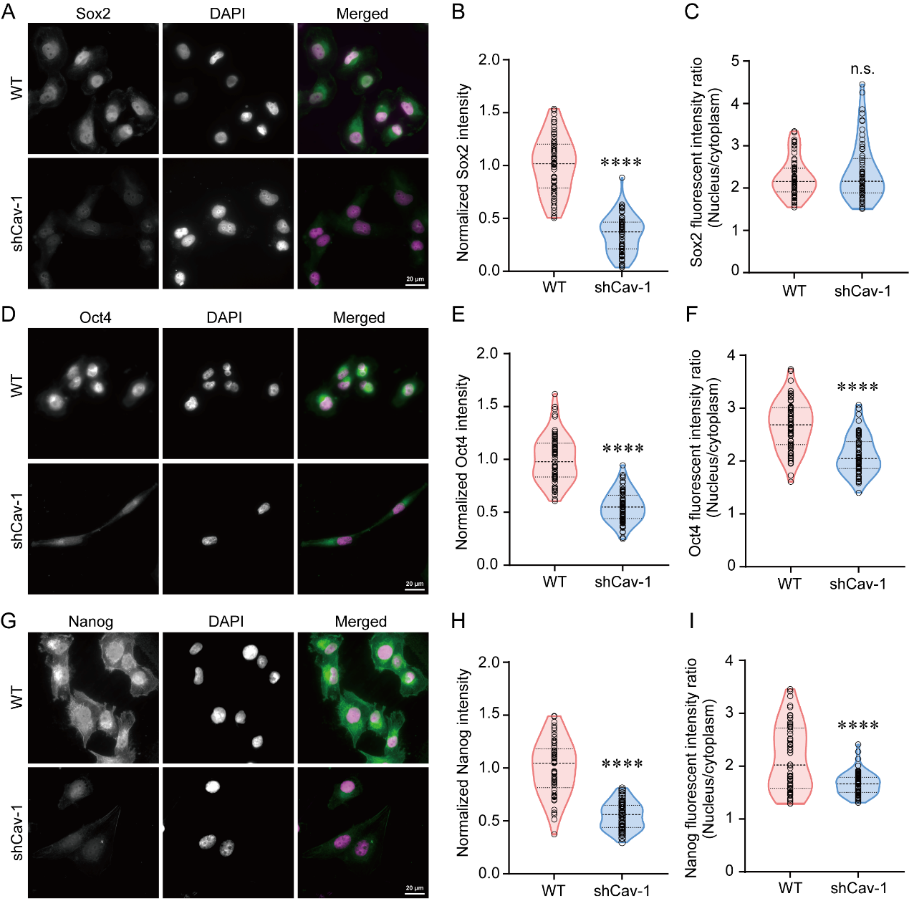


**Figure S3**. (**A**/**D**/**G**) Immunofluorescence images showing Nanog (A), Sox2 (D) and Oct4 (G) (green) in WT and shCav-1 cells, with nuclei stained with DAPI (magenta in merged images; right column). Scale bar = 20 μm. (**B**/**E**/**H**) Violin plot of quantified Nanog (B), Sox2 (E) and Oct4 (H) levels from images (A/D/G), respectively. **** *p*<0.0001. (**C**/**F**/**I**) Nanog (C), Sox2 (F) and Oct4 (I) distribution from (A/D/G) were represented as the ratio of nuclear-to-cytosolic intensities. **** *p*<0.0001. (B and C) n = 72 in WT group and n = 60 in shCav-1 group, (E and F) n = 75 in WT group and n = 72 in shCav-1 group, (H and I) n = 64 in WT group and n = 70 in shCav-1 group.


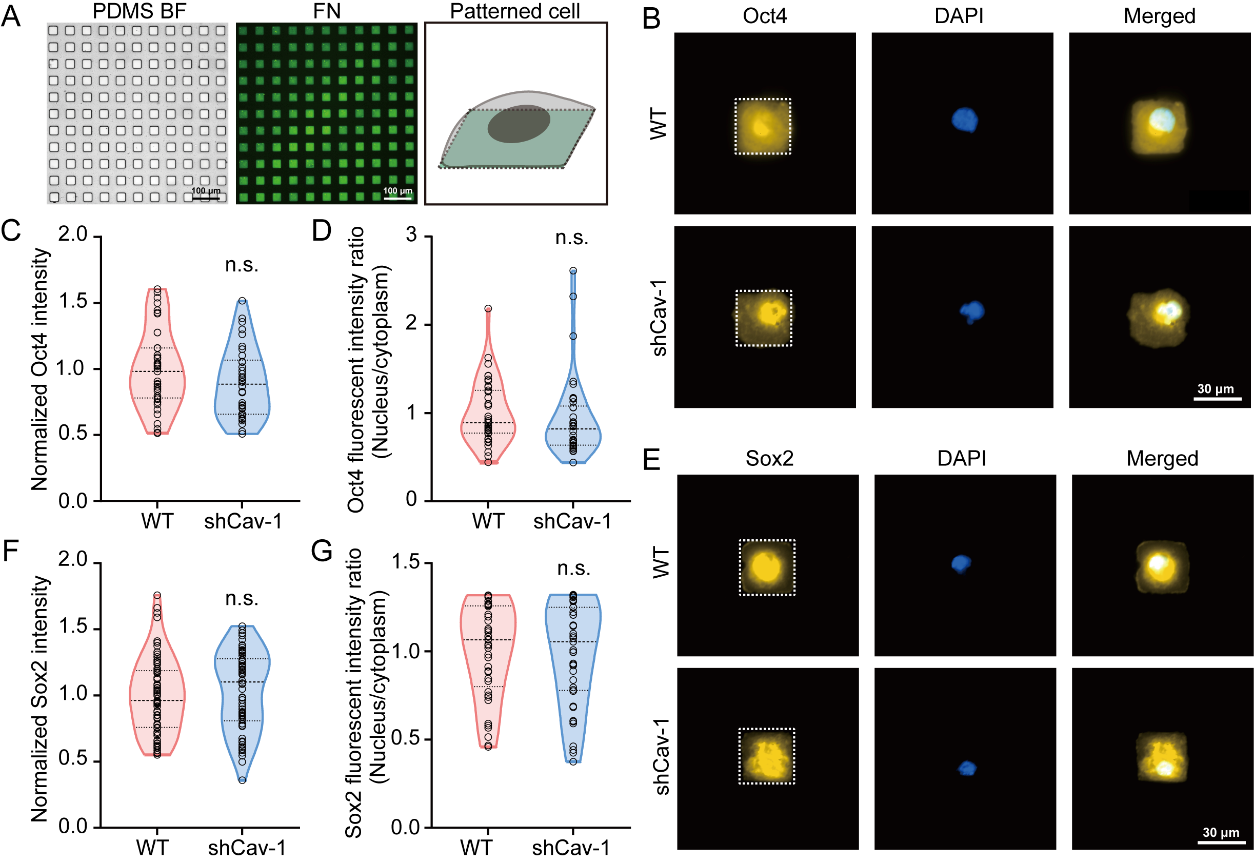


**Figure S4**. (**A**) Bright field of the PDMS mold (left), green fluorescence channel picture of the culture dish after micropattern printing (middle), and schematic diagram of cells seeded on the micropattern (right). Scale bar = 100 μm. (**B**/**E**) Immunofluorescence images showing Oct4 (B) and Sox2 (E) (yellow) in WT and shCav-1 cells seeded on micropatterns, with nuclei stained with DAPI (blue in merged images; right column). Scale bar = 30 μm. (**C**/**F**) Violin plot of quantified Oct4 (C) and Sox2 (F) levels from images (B/E), respectively. (**D**/**G**) Oct4 (D) and Sox2 (G) distribution from (B/E) were represented as the ratio of nuclear-to-cytosolic intensities. (C and D) n = 39 in WT group and n = 38 in shCav-1 group, (F and G) n = 79 in WT group and n = 70 in shCav-1 group


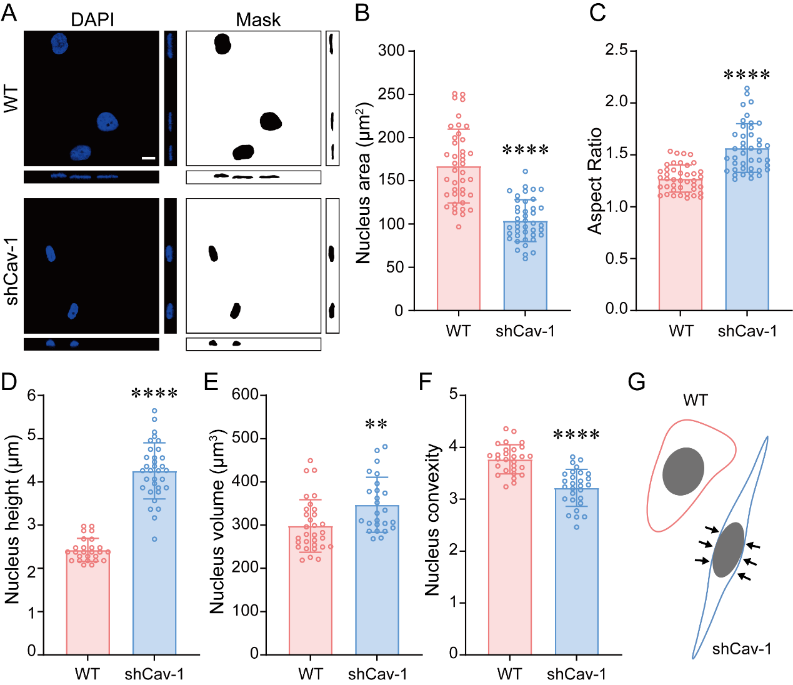


**Figure S5**. (**A**) Images of nuclei in WT and shCav-1 cells stained with DAPI (blue, left column) along with their corresponding nuclear mask images (right column) in the x-y, x-z, and y-z planes. (**B**-**F**) Quantification of the nucleus cross-sectional area (B), nucleus aspect ratio (C), nucleus height (D), nucleus volume (E) and nucleus convexity (F) of WT and shCav-1 cells in (A), ***p*<0.005, *****p*<0.0001. (B-C) n = 41 in WT group and n = 42 in shCav-1 group, (D) n = 26 in WT group and n = 33 in shCav-1 group, (E) n = 31 in WT group and n = 25 in shCav-1 group, (F) n=28. (**G**) Schematic depiction of the cellular and nuclear shapes of WT and shCav-1 cells.

**Movie Legend**

**Movie 1.** The computational simulation of cancer cell morphological evolution. Time-lapse sequence of cancer cell morphological expansion which includes 3 steps: isotopic expansion, A-P polarization and depolarization at the indicated different backgrounds. Green color marks the cell boundary at basal domain. The unit of time is seconds.
